# Airway Management of Patients with Suspected or Confirmed COVID-19: Survey Results from Physicians from 19 Countries in Latin America

**DOI:** 10.3390/jcm11164731

**Published:** 2022-08-12

**Authors:** Manuel Granell, Nerea Sanchis, Carlos Delgado, Manuel Lozano, Marcio Pinho, Cecilia Sandoval, Carolina S. Romero, Cesar Aldecoa, Juan P. Cata, Jorge Neira, Jose De Andres, Alejandro Herreros-Pomares, Guillermo Navarro, The COV2-VIAEREA Network Study Group

**Affiliations:** 1 Department of Surgery (Anesthesiology), Faculty of Medicine, University of València, 46010 Valencia, Spain; 2 Department of Anesthesia, Critical Care and Pain Medicine, Consortium València General University Hospital of València, 46014 Valencia, Spain; 3 Faculty of Pharmacy, University of València, 46010 Valencia, Spain; 4 Department of Anesthesia, Critical Care and Pain Medicine, Central Hospital of the Military Police of Rio de Janeiro, Rio de Janeiro 20221-270, Brazil; 5 Department of Anesthesia, Critical Care and Pain Medicine, Hospital Angeles León, Leon 37150, Mexico; 6 Research Department, European University of València, 46010 Valencia, Spain; 7 Department of Anesthesia, Critical Care and Pain Medicine, University Hospital Río Hortega of Valladolid, 47012 Valladolid, Spain; 8 Department of Anesthesia, Critical Care and Pain Medicine, Anderson Cancer Center, Houston, TX 77030, USA; 9 Trauma Foundation Buenos Aires, Buenos Aires 1071, Argentina; 10 Research Foundation of the University General Hospital of València, 46014 Valencia, Spain; 11 Department of Anesthesia, Critical Care and Pain Medicine, Emergency Hospital Dr. Clemente Alvarez, Rosario S2000, Argentina

**Keywords:** airway management, COVID-19 patients, tracheal intubation, airway devices, video laryngoscope, cross-infection, Latin American countries

## Abstract

Airway management during the COVID-19 pandemic has been one of the most challenging aspects of care that anesthesiologists and intensivists face. This study was conducted to evaluate the management of tracheal intubation in patients with suspected or confirmed COVID-19 infection. This is a cross-sectional and international multicenter study based on a 37-item questionnaire. The survey was available to physicians who had performed intubations and tracheostomies in patients with suspected or confirmed COVID-19 and had provided informed consent to participate. The primary outcome is the preference to use a specific device for tracheal intubation. Secondary outcomes are clinical practice variables, use of video laryngoscopes, difficult airway management, and safety features to prevent cross-infection. This study included 2411 physicians who performed an average of 11.90 and 20.67 tracheal intubations in patients diagnosed or suspected of having COVID-19 disease, respectively. Physicians were mainly from the specialties of Anesthesiology (61.2%) and Intensive Care (7.4%). COVID-19 infection diagnosed by positive PCR or serology in physicians participating in intubation in this study was 15.1%. Respondents considered preoxygenation for more than three minutes very useful (75.7%). The preferred device for tracheal intubation was the video laryngoscope (64.8%). However, the direct laryngoscope (57.9%) was the most commonly used, followed by the video laryngoscope (37.5%). The preferred device to facilitate intubation was the Eschmann guide (34.2%). Percutaneous tracheostomy was the preferred technique (39.5%) over the open tracheostomy (22%). The predicted or unpredicted difficult airway management in these patients was preferably performed with a video laryngoscope (61.7% or 63.7, respectively). Intubation was mostly performed by two or more expert airway physicians (61.6%). The use of personal protective equipment increased the practitioners’ discomfort during intubation maneuvers. The video laryngoscope is the preferred device for intubating patients with COVID-19, combined with the Eschmann guide, flexible stylet within the endotracheal tube, or Frova guide to facilitate intubation. The sub-analysis of the two groups of physicians by the level of intubation experience showed a higher use of the video laryngoscope (63.4%) in the experts group and no significant differences between the two groups in terms of cross-infection rates in physicians, in their preference for the use of the video laryngoscope or in the number of intubations performed in confirmed or suspected COVID-19 patients.

## 1. Introduction

Severe acute respiratory syndrome coronavirus 2 (SARS-CoV-2), also called COVID-19, was declared a pandemic by the World Health Organization (WHO) in March 2020. Globally, 318,648,834 confirmed cases of COVID-19, including 5,518,343 deaths, were reported to WHO on 14 January 2022 [1]. Non-invasive ventilation (NIV), high-flow nasal cannula (HFNC) oxygen, and prompt endotracheal intubation following NIV failure have been recommended to manage by well-established guidelines on acute respiratory distress syndrome (ARDS) treatments [2].

In a series of 1099 patients with laboratory-confirmed COVID-19 in China during the first two months of the current outbreak, 5.0% required ICU admission, 2.3% were placed on invasive mechanical ventilation, and 1.4% died [3]. The WHO estimated 80,000 and 180,000 health and care workers (HCW) could have died from COVID-19 between January 2020 and May 2021, converging to 115500 deaths [1], with a 5.62% infection rate among HCW, and emergency rooms had the highest rate (30.6%) of infection [4]. Hence, cross-infection between healthcare professionals and infected patients is another risk that leads to wearing appropriate personal protective equipment (PPE) [5].

The cause–effect relationship between the performance of intubation and SARS-CoV2 infection is unknown. Thus, several devices designed to prevent the spread of the virus during airway manipulation, such as the intubation box, were used but have since been largely discarded. The WHO reported that tracheal intubation, non-invasive positive pressure ventilation, tracheostomy, cardiopulmonary resuscitation, bronchoscopy, and sputum induction (e.g., respiratory physiotherapy) are aerosol-generating procedures (AEPs) associated with a significant risk of infection in HCWs [6].

Healthcare workers who perform tracheal intubations have a three to six times higher risk of becoming infected than the others [7]. The leading causes favoring this cross-infection during intubation maneuvers are the proximity of the physicians to the infected patient’s airway and face-mask-assisted manual ventilation with positive pressure before intubation. Patient-related factors (e.g., severe illness, high viral load, cough, heavy breathing, and super-spreaders), forced airflows, and prolonged exposure during procedures (e.g., tracheostomy) should also be highlighted [8]. The indication for tracheal intubation in patients with COVID-19 in critical respiratory failure has changed during the evolution of the pandemic. The synthesized evidence suggests that the timing of intubation may not affect the mortality and morbidity of critically ill patients with COVID-19. These results might justify a wait-and-see approach, leading to fewer intubations. Relevant guidelines may therefore need to be updated [9].

Clinical guidelines are published for the safe management of these patients, but healthcare workers have been forced to work under challenging circumstances that have often conditioned their care work. Therefore, the Spanish Society of Anesthesiologists (SEDAR) and the Confederación Latinoamericana de Sociedades de Anestesiología (CLASA) promoted a survey aimed at obtaining information on the airway management of patients with COVID-19, particularly on difficulties and risks faced by physicians at the time of endotracheal intubation in daily practice under increased pressure caused by COVID-19 in Latin American countries.

## 2. Materials and Methods

### 2.1. Study Design

The Ethical committee approved this international, multicenter, cross-sectional, prospective, observational survey of Consorcio Hospital General of València, Spain (registration number CPMP/ICH/135/95, on 24 April 2020, with the identification code of “The COV2-VIAEREA Network Study Group”). The study was designed and conducted according to the declaration of Helsinki and was registered at https://clinicaltrials.gov (NCT04487977) and it was accessed on 27 July 2020. A scientific committee formed by four expert anesthesiologists from Spain (SEDAR) and Latin America (CLASA) societies was responsible for the design of the first draft of the study questionnaire. Its content was subsequently refined and expanded according to the opinion of six consultant experts from both scientific societies. Additionally, the first draft was piloted among nine external first-line clinicians from all potential interest groups, including two anesthesiologists, two intensivists, two emergency medicine physicians, one cardiologist, one pulmonologist, and one physician in ambulance services. All of them with at least five years of experience. In addition to airway technical questions, all were requested to improve language and overall understanding of the survey instrument questions. The final questionnaire was approved by consensus through a Delphi method and included 37 items, it should be completed anonymously, and the response to all items was compulsory. Most items were single-answer questions, but some 6-point Likert scale questions (rating from 1 = strongly disagree to 6 = strongly agree) were also included.

Pretesting or pilot testing can significantly enhance the effectiveness of any survey. The present study was performed in two phases. First, the leading research team reviews all aspects of the survey (i.e., how long the survey should take to complete, the instructions, the question’s order, and whether specific questions are ambiguous and are being consistently missed, repeated, among others). Second, the survey was distributed to a group of 10 anesthesiologists of the COV2-VIAEREA NetWorking group before sending it to the larger target group to analyze difficulties or problems.

Data were collected using an electronic survey form through the Internet platform from 28 April to 31 October 2020. The questionnaire was hosted on Microsoft Forms O365 (Microsoft, Redmond, WA, USA). The primary outcome was the preference for using a specific airway device for intubation. Secondary outcomes were clinical practice variables, use of video laryngoscopes, complex airway management, and safety items to prevent infection. This study has also tried to evaluate some characteristics of the participating physicians about their age, experience, number of intubated COVID-19 patients, and COVID-19 infection, among others. The final questionnaire was obtained by consensus and included 37 items, and the response to all items was compulsory. Most of the items were questions in which only one valid answer was chosen, but 6-point Likert scale questions (from 1 = strongly disagree to 6 = strongly agree) were also included. We calculated the percentage of physicians who selected value 6 (maximum agreement). The questionnaire is shown in the Supplementary Materials.

The survey link was distributed by mailing and through official social networks of the Latin American societies directly and exclusively to physicians who could have participated in the tracheal intubation of COVID-19 patients (Anesthesiology, Intensive and Critical Care, Emergency Medicine, Cardiology, Pulmonology, and Thoracic Surgery, and Internal Medicine), excluding other health professionals who have participated in manipulation maneuvers of the airway. SEDAR and CLASA were the initial promoters of this study in Latin America. Particularly active in the dissemination of the survey was EVALa (programa de Entrenamiento en Vía Aérea Latinoamérica/Latin American Airway Training programme) as the airway section of CLASA represented in all Latin American countries. Other health professionals were not taken into account in the distribution of the survey. In addition, at the beginning of this survey, the participants were required to indicate to which medical specialty they belonged and in which Latin American country they were working during that period. Members of SEDAR participated in the design and conduct of the study. Still, Spanish physicians were not invited to participate in the survey intended only for physicians from Latin American countries.

A non-discriminatory exponential snowball sampling was used, by means of which the invitation was sent by the participating societies to the members of the scientific societies, who reinforced this invitation through their professional contacts of the specialties participating in the study until reaching 2000–2500 expected respondents. The goal was to recruit 5–10% of all physicians from the designated medical fields potentially involved in airway management of COVID-19 patients (2000–2500 participants from these 19 countries). This number was estimated from the SEDAR and CLASA Societies and Intensive Care Medicine data, representing 5–10% of physicians in each Latin American country. Countries that did not obtain a minimum response rate (5%) were excluded. Only physicians with Internet access to the Microsoft Forms platform were eligible. Participation was voluntary and unpaid. Informed consent was provided as this requirement had to be completed in the first item of the questionnaire.

### 2.2. Statistical Analysis

Categorical variables were expressed as frequencies and percentages and quantitative variables as mean and standard deviation (SD) or median and range according to the normality test. One-way analysis of variance (ANOVA) and Tukey’s test for multiple comparisons was used for the analysis of the Likert categories ‘never forgetting’ and ‘a few times’ as well as ‘almost always’ and ‘always’ for the variables ‘discomfort with PPE’ and ‘forgetting safety steps’. The variable ‘seniority’ referred to years of experience and was assessed as a continuous variable to compare ‘preferred video laryngoscope’, ‘device most used’, ‘preferred type of blade’, ‘blade most commonly used’, and ‘number of experts during intubation’. The variables ‘number of intubations performed’ and ‘infection’ were analyzed with the Kruskal–Wallis test to determine if there were statistically significant differences between two or more groups of an independent variables on a continuous or ordinal dependent variable, such as in the case of number of intubations that is ordinal, and infection that was coded as an ordinal variable with four levels. Statistical significance was set at *p* < 0.05. The R Statistical Program (version 3.5.2, R Core Team, Vienna, Austria) was used to analyze data.

Normality was test with Shapiro–Wilk test, where a *p* value > 0.05 was considered the cutoff point to name a variable normally distributed.

Descriptive analysis was performed with ANOVA of the respondents’ demographic data (age and seniority) and the number of patients intubated with confirmed COVID-19 or suspected COVID-19. This information helps us to put into context some characteristics of the workforce serving intubations in COVID-19 patients and know the number of techniques performed in each clinical scenario. We did not control for multiple testing because we are not testing simultaneously in the same outcome.

A *X*^2^ test was performed using SPSS 20.0 version (Statistical Package for the Social Sciences, Chicago, IL, USA) to check whether airway expertise was related to the number of confirmed or suspected COVID-19 intubated patients, COVID-19 cross-infection rates in physicians, or any specific intubation devices as the most preferred or used among professionals. Anesthesiologists and critical care physicians with over ten years of experience were considered to be airway experts.

## 3. Results

This study included 2411 physicians from 19 Latin American countries who performed tracheal intubations in patients diagnosed or suspected of having COVID-19 disease. The physicians were mainly from the specialties of Anesthesiology and Intensive Care. Fifteen of the physicians participating in this study had a COVID-19 infection diagnosed by PCR or serology during this study period. The preferred device for tracheal intubation was the video laryngoscope, but the most commonly used device was the direct laryngoscope, followed by the video laryngoscope. The preferred device to facilitate intubation was the Eschmann’s guide. Respondents found preoxygenation for more than three minutes beneficial. The anticipated or unanticipated complex airway management in these patients was preferably performed with a video laryngoscope.

**Figure 1:**
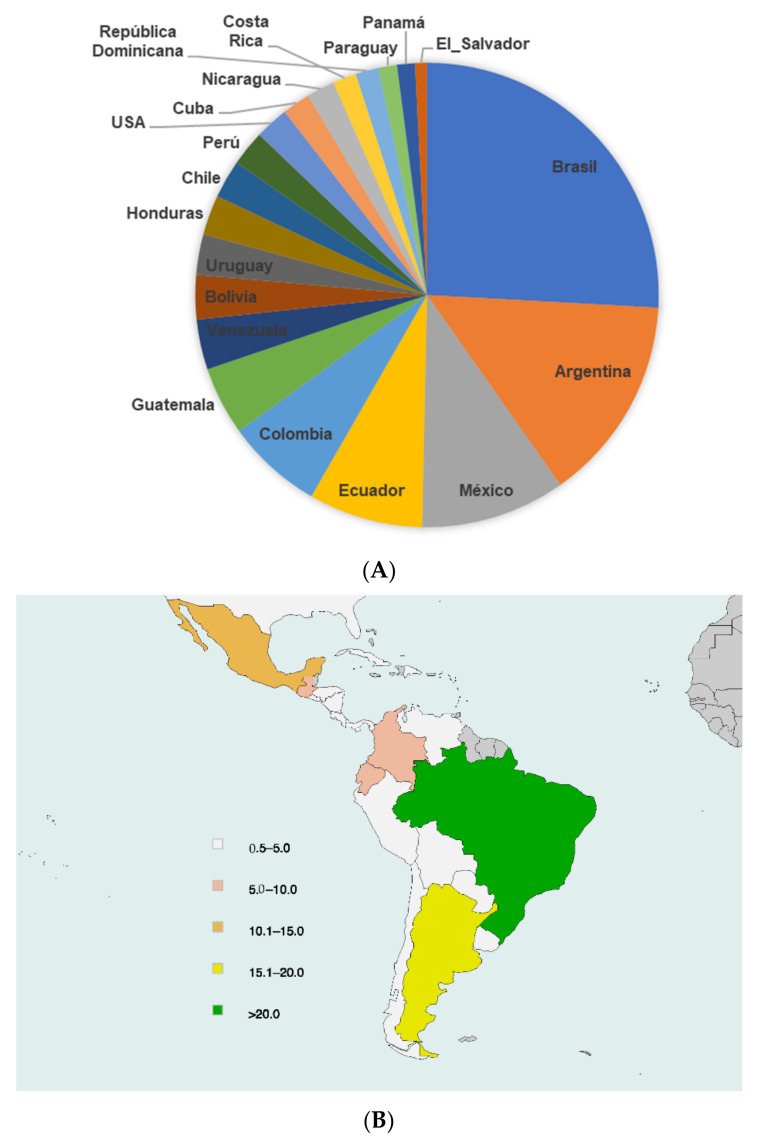
(**A**) Relative percentage of participating members of every country from Latin America. (**B**) Percentage of participating members from each country in relation to the total number of participants from all Latin American countries.

**Figure 2:**
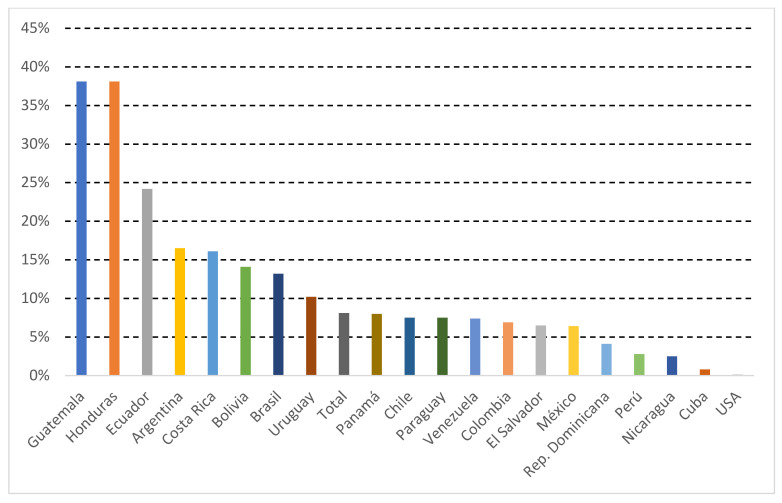
Participating members/Total Membership from each country (%). United States of America (USA). Republic (Rep).

The highest rate of physicians completing the survey were those from Brazil (27.4%), Argentina (15.2%), and Mexico (10.7%) (Figure 1A,B). In most countries (16/19), participation was higher than 5% of the national membership of anesthesiologists (Figure 2).

The participants had a mean age of 41.91 years and a standard deviation (SD) of 10.26. They had an average practicing experience of 21.19 years (±16.88) (Table 1). The survey was answered mainly by anesthesiologists (61.2%) and also critical care/ intensive care specialists (7.4%) (Table 2). Among all respondents, the mean number of tracheal intubations was 11.90 (21.18) and 20.67 (40) in patients with COVID-19 positive and suspected cases, respectively (Table 1). The most common workplace where physicians performed tracheal intubation of patients with COVID-19 was public hospitals (46%), and the most frequent location where tracheal intubations were performed is shown in Table 2.

On the other hand, COVID-19 infection diagnosed by positive PCR in physicians participating in intubation in this study was 15.1%; 55.9% of cases were confirmed by PCR, while 29% did not undergo any diagnostic test during the study because they were asymptomatic (Table 2).

**Figure 3:**
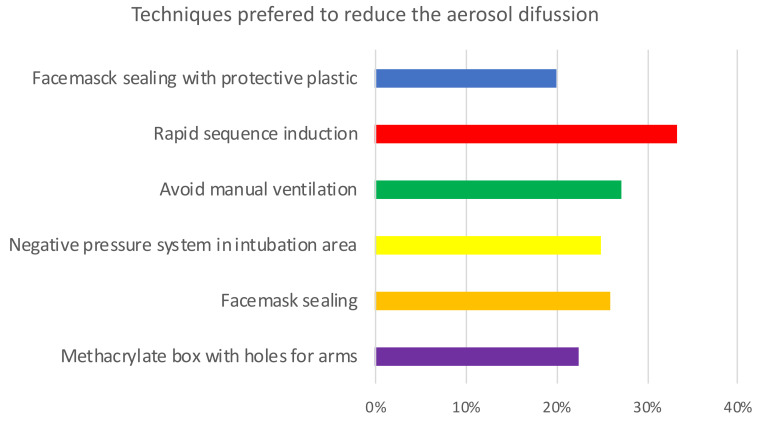
Opinion on the usefulness of techniques to reduce aerosol diffusion during tracheal intubation in COVID-19 patients. 6-point Likert scale (from 1 = strongly disagree to 6 = strongly agree; Percentage scoring 6: n (%).

**Figure 4:**
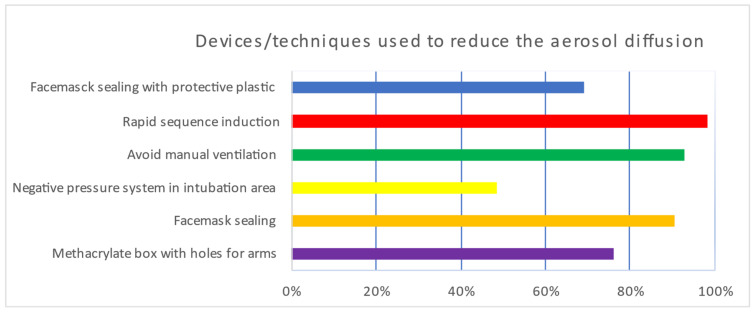
Used techniques to reduce aerosol diffusion during tracheal intubation in COVID-19 patients. Percentage (%).

Table 3 and Table S1 show the preferred methods to perform the airway management in COVID-19 patients. Regarding the preferred methods to avoid aerosolization, rapid sequence induction (RSI) was first, followed by the avoidance of manual ventilation, face mask sealing, negative pressure areas, the use of methacrylate box, and plastic covering (Table 3, Figure 3). However, the Sellick’s maneuver was the least preferred technique (18%) for intubation support. Regarding methods used to reduce aerosol production, it was found that >90% of respondents used RSI, face mask sealing, and avoided manual ventilation; 76% used methacrylate boxes and 69% used plastic covering for intubation; 48.7% were intubated in areas with negative pressure; the RSI technique was most likely performed to intubate COVID-19 patients (98.4%) and 64.5% of respondents applied cricoid force (Sellick´s maneuver) to prevent pulmonary aspiration (Figure 4).

**Figure 5:**
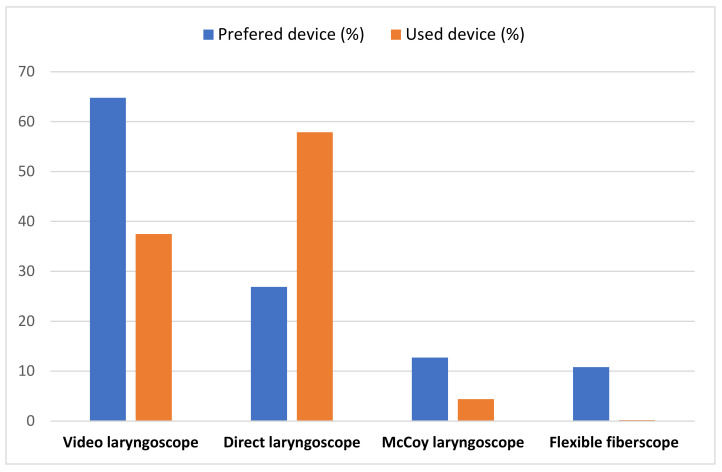
Percentage of the most preferred or used airway devices to intubate COVID-19 patients.

Respondents’ opinions on device preference indicated that the optimal device for intubation in patients with COVID-19 was the video laryngoscope (64.8%), followed by the direct laryngoscope (26.9%) (Table 3, Figure 5). The preferred type of video laryngoscope was as follows in descending order: C-MAC, King Vision, McGrath, Glidescope, and Airtraq (Figure S1); other laryngoscope characteristics are described in Table 3. Moreover, for the most preferred devices to facilitate intubation, the participants chose in decreasing order: Eschmann guidewire, flexible stylet inside the endotracheal tube, Frova guidewire, fiberscope, and finally, the VAMA cannula (Table 3).

Among the preferred devices for tracheal intubation in cases of predicted or known difficult airway in patients with COVID-19, the video laryngoscope was chosen (61.7%), followed by the fiberscope (31.6%) (Table 3). The respondents’ preferred tracheostomy technique was percutaneous versus open tracheostomy (Table S1). Intubation while wearing PPE was very uncomfortable for the staff who performed it; moreover, intubation was associated with increased difficulty in performing the technique (Table S1). Respondents preferred extubation in the operating theatre after surgery when patients were stable and might not require postoperative mechanical ventilation (66.2%) (Table S1).

Table 4 shows the methods used to perform airway management in COVID-19 patients. The use of pre-oxygenation before intubation as a clinical safety measure was applied by the respondents for 3–5 min (46.3%) or more than 5 min (29.3%). However, it could not be used in 10.7% of cases because of the patient’s conditions or the stressful context (Table 4). In addition, the number of physicians participating during the intubation was assessed in our survey. In this study, 61.6% of participants informed that two or more had experienced during intubation (Table 4). Regarding following the safety steps/sequence for performing the intubation maneuver, 69.6% indicated they had missed some safety steps. In comparison, 17.2% indicated they almost always missed some of the necessary safety steps. In the last question related to clinical safety, it was found that what caused the most significant stress to physicians was the risk of being infected (38%), followed by the possibility of encountering a difficult airway and intubation failure and patient deterioration (Table 4).

We investigated which techniques and devices had been most commonly used for intubation during this period in Latin America for patients with COVID-19 (Table 4). The most widely used device for the tracheal intubation of these patients was the direct laryngoscope (57.9%), followed by the video laryngoscope (37.5%) (Figure 5). The video laryngoscope, the most commonly used by physicians, was the following in decreasing order: McGrath, C-MAC, King Vision, Glidescope and Airtraq. However, 27.9% of the participants indicated that they did not have a video laryngoscope in their work area, while 16.8% answered that they intubated with a direct laryngoscope although they had a video laryngoscope; the most commonly used video laryngoscope blades were reusable (47.4%) versus disposable (26.5%) (Table 4).

Finally, one of the most important contributions of this study is the sub-analysis performed to check whether airway expertise was related to any specific intubation devices as the most preferred or used among professionals or with COVID-19 infection rates among airway experts or not airway experts professionals. Anesthesiologists and critical care physicians with over ten years of experience were considered to be airway experts. Results are shown in Tables S2–S4 and Figures S2 and S3.

The results of the sub-analysis between the two groups according to their airway management expertise showed that video laryngoscopes were used more frequently by airway experts than in the non-expert group (*p* < 0.00001) (Table S2, Figure S2). There was no statistically significant association between airway expertise and the preferred device to perform intubations (Table S3). It was found that there were no statistically significant differences between the number of intubations of confirmed or suspected COVID-19 patients between both groups (18.1 ± 9.0 in non-experts, 21.4 ± 12.1 in experts; *p* = 0.214).

These sub-analysis results also show a similar COVID-19 infection rate among the physicians from the experts group (14.9%) and the non-experts group (16.3%) (Table S4, Figure S3).

## 4. Discussion

This study involved 2411 physicians who performed an average of 11.90 and 20.67 tracheal intubations in patients diagnosed or suspected of having COVID-19 disease, respectively. Fifteen percent of the physicians participating in this study had a COVID-19 infection diagnosed by PCR or serology during this study period. The physicians were mainly from the specialties of Anesthesiology and Intensive Care who are familiar with multiple airway devices. Thus, the previous experience of the clinicians with the devices and techniques evaluated influenced the outcomes of this study. Respondents found preoxygenation for more than three minutes beneficial. The preferred device for tracheal intubation was the video laryngoscope, but the most commonly used device was the direct laryngoscope, followed by the video laryngoscope. The preferred device to facilitate intubation was the Eschmann’s guide. Percutaneous tracheostomy was the preferred technique over open tracheostomy. The anticipated or unanticipated difficult airway management in these patients was preferably performed with a video laryngoscope. Two or more experienced airway physicians mostly performed intubation.

Our study demonstrated that the incidence of COVID-19 infection among participating physicians was 15.1%. On the one hand, this rate of infection is slightly higher than in the other two previous studies, including one that was performed in Spain (11.6%) [10] and another (10.7%) conducted in 17 countries during the initial period of the pandemic (March–June 2020) [11]. On the other hand, some participating physicians did not undergo this diagnostic test during this period, so this incidence may be underestimated. Other studies confirm that at least 3% of asymptomatic healthcare professionals had positive results when the PCR test was performed systematically [12].

The use of pre-oxygenation prior to intubation as a clinical safety measure for 3–5 min (46.3%) or more than 5 min (29.3%), according to previous recommendations [10]. Nevertheless, 10.7% of physicians could not apply these measures because of the patient’s conditions or the stressful context (Table 4). Nowadays, experts recommend using 5 min of preoxygenation with 100% oxygen and RSI techniques to avoid the manual ventilation of COVID-19 patient’s lungs and the potential aerosolization of the virus from airways [13]. Moreover, recent guidelines published for the management of tracheal intubation in critically ill patients recommend using high-flow nasal oxygen therapy (HFNO) during and between tracheal intubation attempts [14]. On the other hand, the Difficult Airway Society (DAS) has suggested that high-flow nasal oxygen therapy (HFNO) may have a role in managing a difficult airway [15].

In the present study, more than 90% of respondents had used RSI, face mask sealing, and avoided manual ventilation to reduce aerosol production. In contrast, they used methacrylate boxes and plastic covering for intubation less frequently. Sellick´s maneuver was used less often to avoid lung aspiration. Other studies also propose avoiding coughing maneuvers [16]. Probably, the lower use of Sellick´s maneuver may lie in the evidence that it may hinder tracheal intubation [17,18]. The low positive assessment regarding the use of the aerosol intubation box may be related to the fact that its use is associated with a longer time required for tracheal intubation and less mobility and visibility of the professional performing it. In addition, it has been reported that it is not uncommon for the PPE seal to break when using the aerosol intubation box and increase intubation time [19,20].

Video laryngoscopy was the preferred method for the intubation of patients in this study. This is consistent with recommendations in most publications to use video laryngoscopy for the intubation of COVID-19 patients due to the lower aerosol exposure for the intubating physician, as the practitioner’s head is further away from the patient’s airway [10,21]. The most likely explanation is that video laryngoscopes improve the glottis’s vision, increase the success rate at the first attempt, reduce the force required for intubation, and reduce complications on the airway compared to direct laryngoscopy [22,23]. However, we observed that the most commonly used device for intubating patients with COVID-19 was a direct laryngoscope. This discrepancy between preference and actual use may be related to the unavailability of video laryngoscopes in many centers and their preference based on experience. They reported that approximately 50% had never used the video laryngoscopes included in the survey. However, this contrasts paradoxically with the fact that 27.9% of physicians did not have any video laryngoscope available to intubate COVID-19 patients; in addition, 16.8% of participants preferred to use direct laryngoscopy even if they had video laryngoscopes and 28.6% indicated that the most appropriate device for this intubation was the direct laryngoscope. All this probably means that there was a limitation of resources and that some clinicians did not have the optimal skills for its use, so they preferred to perform direct laryngoscopy.

Thus, in this study conducted in Latin America, a greater use of direct laryngoscopy was observed (57.9%) compared to the majority use of video laryngoscopes observed in other areas of the world for the intubation of COVID-19 patients, which ranged between 70.5% [10] and 76.1% [11]. In these last two studies, the incidence of COVID-19 infection was 11.7% [10] and 10% [11], while in the conducted exclusively in Latin America, it was much higher (15.1%). This difference in the infection rate may be related to the use or not of the video laryngoscope since it allows health personnel to be kept away from the COVID-19 patient’s airway. In some of these studies, it was also shown that physicians who intubated patients with direct laryngoscopy in out-of-hospital areas (90% of cases) had higher rates of COVID-19 infection (17.5%) than other professionals who generally intubated using a video laryngoscope (11.7%) [10]. This should lead us to recommend more strongly the future use of video laryngoscopy in patients diagnosed with or suspected of having COVID-19 disease.

The preferred video laryngoscope blades were disposable as recommended by guidelines [22,23] and published studies.^11^ However, the reusable video laryngoscope blade was the most frequently used compared to the disposable type, probably due to the lack of availability in many healthcare centers during the study period. In agreement with a previous study, the most preferred video laryngoscope blade curvature was the MacIntosh [10]. Moreover, the video laryngoscope blade with a channel was preferred, although the blade without a channel was also highly rated. In addition, respondents showed no differences about the preference for using video laryngoscope monitors attached or remotely, although this contrasts with expert recommendations [22,24].

Regarding the preferred adjuncts to devices to facilitate intubation, they indicated an interest in using the Eschmann guide, the flexible stylet within the endotracheal tube, the Frova guide, or the fiberscope within the endotracheal tube. Previous publications suggest the suitability of the combined use of the video laryngoscope and a bougie/facilitator guide, such as the Eschmann or Frova guides included in this questionnaire, as they obtained an approximate success rate of 98% [25]. Other authors have found that using the flexible stylet inside the orotracheal tube facilitates intubation when a 60° curvature is applied since it increases intubation speed [22,26]. The least used device was the fiberscope, which agrees with the recommendations [6]. To manage expected or known difficult airways in patients with COVID-19, the respondents preferred to use a video laryngoscope, followed by a fiberscope and direct laryngoscope. These results align with the published guidelines [22] and are consistent with previously published studies [10].

Tracheostomy in patients with COVID-19 is considered one of the surgical procedures that poses the highest risk of infection for healthcare workers. In agreement with other authors, most participants in this study indicated a preference for percutaneous tracheostomy over open tracheostomy [27]. The preference can be explained by its shorter duration, less aerosolization, and the fact that it can be performed at the ICU bedside [28]. Using an apnea period during tracheostomy to avoid aerosol formation was preferred by most of the participants in this study. However, the response was heterogeneous and the difference was not statistically significant. Nevertheless, the recommendation seems appropriate and is consistent with the publications reviewed on the subject [29].

The importance of using PPE to reduce patient/clinician cross-infection was addressed regarding the strategy to improve clinical safety during intubation. Using PPE can markedly reduce the infection risk associated with caring for COVID-19 patients [30]. Although there is little evidence on which PPE offers the best protection, improved training in PPE, likely donning and doffing, simulation, and instructions for optimal PPE handling, would be beneficial [31]. However, some studies report some drawbacks to the use of PPE during intubation; thus, the success rate of intubation with direct laryngoscopy or video laryngoscopy on the first attempt has been reported to be 96% without PPE, while it is reduced to 58% when PPE is used [25].

It is important to consider the number of health care providers necessary to intubate COVID-19 patients as a measure of safety. Three individuals are likely required: an intubator, an assistant, and a third person to give drugs and watch monitors [23]. In our study, 61.6% of the responders reported two or more physicians as participants during the intubation, similar to the current recommendations [23]. Additionally, the frequency in which some safety step was breached was analyzed. A total of 69% of the participants indicated that they had done so on some occasion, and 17.2% reported that they almost always breached safety measures. This highlights the difficulty of strictly following the protocols in these circumstances.

The most significant stress for physicians was the risk of infection, followed by the possibility of encountering a difficult airway, intubation failure, and the consequent possible deterioration of the patient. Finally, respondents preferred extubation in the operating theatre after surgery when patients were stable, and there was no anticipated need for postoperative mechanical ventilation (Table 4). However, this difference was not statistically significant.

Finally, exciting results were obtained with this sub-analysis to check whether airway expertise was related to specific intubation devices as the most preferred or used or with COVID-19 infection rates among professionals. The video laryngoscopes were used more frequently by airway experts than in the non-experts group, possibly due to their deeper knowledge and skill in the usage of these devices. Previous experience with the video laryngoscope device in day-to-day practice should therefore be highly valued. The learning curve for video laryngoscopes may be relevant [32]. Nevertheless, both groups prefer to use video laryngoscopes according to the published guidelines [10,21]. This contradiction is fundamental because it seems necessary to apply theoretical and practical training strategies to improve the confidence of non-experts for the widespread use of video laryngoscope for the intubation of such patients at risk of airborne disease contagion.

Probably one of the most important findings of this study is the high infection rate observed in the participants (15.1%), likely associated with the insufficient use of the video laryngoscope for the intubation of COVID-19 patients, a device that is strongly advised by published guidelines [22,23,24]; furthermore, in this sub-study between airway experts and non-experts, it was concluded that this infection rate was similar in both groups. Moreover, there was no statistically significant difference between the number of intubations of confirmed or suspected positive patients between the two groups; probably, the low use of video laryngoscope could be one of the reasons for the higher incidence of COVID-19 in the healthcare workers in this study in Latin America. Still, more clinical trials should be conducted in the future to definitively clarify all the causes for the higher infection rate found in this study.

The results of this survey show that Anesthesiologists (61.2%) and Intensive Medicine specialists (7.4%) were the professionals who participated the most in COVID-19 tracheal intubation. Still, this survey also includes a significant percentage of other specialties (31.4%) involved in intubation. These two specialties of Anesthesiology and Intensive Medicine are usually involved during the tracheal intubation of critical or surgical patients. This result probably does not reflect a bias and shows the reality of the Latin American experience during the first pandemic wave. Probably, this result shown in Table 2 reflects a reality rather than a bias. Most intubations were performed in operating rooms (56.5%), but 43.5% were performed outside the operating room; probably, as in other places around the world, operating theaters were selected to intubate critically ill patients from other areas to the hospital (e.g., in Spain, 24.5% of COVID-19 patients were intubated in operating theaters during the first wave of the pandemic) [10], since these areas are better equipped for safe intubation (e.g., they have negative pressure and complete airway equipment).

This study has limitations. This is a prospective observational study. The survey was designed to analyze the experience of physicians from different specialties in Latin America. Still, it was primarily answered by specialists in Anesthesiology and to a lesser extent in Intensive Care Medicine, although 28.7%% answered belonging to “Other specialties” not specified in this study. The participating respondents performed tracheal intubations in a higher percentage in patients with suspected SARS-CoV-2 (20.67 patients/physician) than with a confirmed diagnosis (11.90 patients/physician). The invitation emphasized that participants in the survey must be physicians from Latin American countries and respond only once. Still, there is no absolute certainty that these conditions have always been met because it is an anonymous survey.

## 5. Conclusions

This study demonstrated that the preferred device for intubation in patients diagnosed or suspected of having COVID-19 disease was the video laryngoscope. However, the direct laryngoscope (57.9%) was the most commonly used, followed by the video laryngoscope (37.5%). The sub-analysis results between the two groups according to their airway management expertise showed that video laryngoscopes were used more frequently by airway experts than in the non-expert group. Additionally, it calls for improving the availability of video laryngoscopes devices in healthcare centers in Latin America and the training required for optimal management of these devices. The most preferred blade type was disposable, with MacIntosh curvature and a channel. The preferred devices to facilitate intubation was the Eschmann guide, flexible in-tube stylet, and Frova guide. Percutaneous tracheostomy was the preferred technique over open tracheostomy. The difficult airway management in these patients was preferably performed with a video laryngoscope. Intubation was performed mainly by two or more physicians with airway expertise. The use of PPE increased the discomfort of the professionals during intubation maneuvers and slightly increased the difficulty in achieving intubation. The incidence of COVID-19 infection (15.1%) was higher than that observed in other studies.

## Figures and Tables

**Table 1:** Characteristics of the physicians who participated in the survey.

Variable	Mean	SD	CI 95 Inf	CI 95 Sup
Age	41, 91	10, 26	41, 5	42, 31
Seniority	21, 19	16, 88	20, 51	21, 86
Number of COVID-19 confirmed intubated patients	11, 90	21, 18	11, 06	12, 75
Number of COVID-19 suspected intubated patients	20, 67	40, 00	19, 08	22, 27

Standard deviation (SD). Confidence interval (CI).

**Table 2:** Profile of the physicians who participated in the survey.

Items of the Questionnaire	n (%)	* *p* Value
Which is the most accurate description of the hospital where you normally work?		<0.001
Public hospital	1109 (46%)	
Private hospital	618 (25.6%)	
Public–private hospital	684 (28.4%)	
What is your medical specialty in where you have had experience with airway management in COVID-19 patients?		<0.001
Anesthesiology	1475 (61.2%)	
Critical Care Medicine	179 (7.4%)	
Emergency Medicine	53 (2.2%)	
Internal Medicine	13 (0.5%)	
Other	691 (28.7%)	
In your clinical experience, where have you intubated more COVID-19 patients?		<0.001
Emergencies/out-of-hospital emergencies	44 (1.8%)	
Hospital emergency department	279 (11.6%)	
Intensive care unit	600 (24.9%)	
Urgent surgery	1002 (41.5%)	
Scheduled surgery	361 (15%)	
Hospitalization ward	125 (5.2%)	
As a front line exposure professional, have you been infected by COVID-19?		<0.001
Yes, I have been diagnosed positive by PCR or serology	365 (15.1%)	
No, I have been diagnosed negative by PCR or serology	1347 (55.9%)	
I have never been tested because I have been asymptomatic	648 (26.9%)	
I have never been tested, although I have had symptoms	51 (2.1%)	

n (Number). % (Percentage). The study questionnaire was distributed among physicians from Latin America. * *p* value (Pearson’s test).

**Table 3:** Preferences in airway management in COVID-19 patients.

Items of the Questionnaire	n (%)	* *p* Value
What do you think about the following systems for the reduction of diffusion of aerosols? 6-point Likert scale (from 1 = strongly disagree to 6 = strongly agree; percentage scoring 6: n (%)).		
Methacrylate box with holes for arms	543 (22.5%)	<0.001
Face mask sealing	622 (25.8%)	<0.001
Negative pressure system in intubation area	601 (24.9%)	<0.001
Avoid manual ventilation	654 (27.1%)	<0.001
Rapid sequence induction	803 (33.3%)	<0.001
Face mask sealing with protective plastic	483 (20%)	<0.001
Based on your COVID-19 patients’ experience, what is the optimal device for intubation? 6-point Likert scale. Percentage scoring 6. n (%).	Number n (%)	
Video laryngoscope	1562 (64.8 %)	<0.001
Direct laryngoscope	649 (26.9%)	<0.001
McCoy laryngoscope	307 (12.7%)	<0.001
Flexible fiberscope	260 (10.8%)	<0.001
What type of video laryngoscopy would you prefer to intubate a COVID-19 patient? 6-point Likert scale. Percentage scoring 6. n (%).		
C-MAC	808 (33.5%)	<0.001
King Vision	629 (26.1%)	<0.001
McGrath	594 (24.6%)	<0.001
Glidescope	562 (23.3%)	<0.001
Airtraq	271 (11.2%)	<0.001
Direct laryngoscope	690 (28.6%)	<0.001
Others	235 (9.7%)	<0.001
In COVID-19 patients, what type of video laryngoscope blade do you prefer to use?		<0.001
Reusable	398 (16.5%)	
Disposable	1191 (49.4%)	
Indifferent	317 (13.2%)	
I do not have experience	505 (20.9%)	
What type of video laryngoscope blade do you prefer to use?		<0.001
With channel	835 (34.6%)	
Without channel	581 (24.1%)	
Indifferent	464 (19.3%)	
I do not have experience	531 (22%)	
In COVID-19 patients, what type of video laryngoscope blade do you prefer to use?		<0.001
Macintosh blade	1234 (51.2%)	
Hypercurved blade	565 (23.4%)	
Indifferent	349 (14.5%)	
I do not have experience	263 (10.9%)	
In COVID 19 patients, what type of video laryngoscope image display monitor do you prefer to use?		<0.001
Video laryngoscope attachment	842 (34.9%)	
Separate/remote from video laryngoscope	856 (35.5%)	
Indifferent	387(16.1%)	
I do not have experience	326 (13.5%)	
What disadvantages of video laryngoscopes consider the most detrimental. 6-point Likert scale. Percentage scoring 6: n (%).		
Annoying light reflections on the video laryngoscope screen	262 (10.9%)	<0.001
Difficulty introducing into the mouth	269 (11.2%)	<0.001
Need for proximity to the patient’s upper airways	311 (12.9%)	<0.001
Difficulty inserting the tube through the vocal cords	291 (12.1%)	<0.001
Lack of practical experience with any of the video laryngoscopes used	589 (24.4%)	<0.001
What type of device do you usually use to facilitate intubation? 6-point Likert scale. Percentage scoring 6: n (%).		
Frova guide	529 (21.6%)	<0.001
Eschmann guide	825 (34.2%)	<0.001
Flexible stylet inside the orotracheal tube	735 (31.9%)	<0.001
Fiberscope	418 (17.3%)	<0.001
VAMA	155 (6.4%)	<0.001
None	124 (5.1%)	<0.001
In the case of a predicted or known difficult airway of a COVID-19 patient, how do you prefer to perform the intubation? 6-point Likert scale. Percentage scoring 6: n (%).		
Flexible fiberscope	761 (31.6%)	<0.001
Video laryngoscope	1487 (61.7%)	<0.001
Direct laryngoscope	475 (19.7%)	<0.001
McCoy laryngoscope	336 (13.9%)	<0.001
Laryngeal mask	153 (6.3%)	<0.001
Tracheal intubation through the laryngeal mask	177 (7.3%)	<0.001
Tracheostomy	175 (7.3%)	<0.001
In the case of unpredicted or unknown difficult airway of COVID-19 patient, how do you prefer to perform the intubation? 6-point Likert scale. Percentage scoring 6. n (%).		
Flexible fiberscope	411 (17%)	<0.001
Video laryngoscope	1536 (63.7%)	<0.001
Direct laryngoscope	581 (24.1%)	<0.001
McCoy laryngoscope	401 (16.6%)	<0.001
Laryngeal mask	252 (10.5%)	0.281
Tracheal intubation through the laryngeal mask	252 (10.5%)	0.066

n (Number). % (Percentage). 6-point Likert scale (from 1 = strongly disagree to 6 = strongly agree; Percentage scoring 6: n (%). * *p* value (Pearson’s test).

**Table 4:** Techniques in airway management of patients with COVID-19 reported by participants.

Items of the Questionnaire	n (%)	* *p* Value
Did you perform preoxygenation prior to intubation/tracheostomy?		<0.001
Yes, for at least 5 min	707 (29.3 %)	
Yes, for 3–5 min	1117 (46.3 %)	
Yes, for less than 1 min	331 (13.7 %)	
No, the patient’s conditions did not allow delay	163 (6.8 %)	
No, context/stress of the situation did not permit us to preoxygenate	93 (3.9 %)	
What is the most frequently used device for intubation in COVID-19 patients?		<0.001
Video laryngoscope	905 (37.5%)	
Direct laryngoscopy	1395 (57.9%)	
McCoy laryngoscope	107 (4.4%)	
Fiberscope	4 (0.2%)	
What kind of video laryngoscope have you used most frequently for intubation in COVID-19 patients?		<0.001
C-MAC	334 (13.9%)	
King Vision	293 (12.2%)	
McGrath	460 (19.1%)	
Glidescope	186 (7.7%)	
Airtraq	59 (2.4%)	
Not available	673 (27.9%)	
They prefer direct laryngoscopy even if they have video laryngoscope	406 (16.8%)	
Please answer “yes” if you have used these video laryngoscopes.	“Yes” n (%)	
C-MAC	1203 (49.9%)	<0.001
King Vision	1207 (50.1%)	<0.001
McGrath	1129 (46.8%)	<0.001
Glidescope	1039 (43.1%)	<0.001
Airtraq	1087 (45.1%)	<0.001
Others	1319 (54.7%)	<0.001
In COVID-19 patients, what type of video laryngoscope blade do you use most frequently?		<0.001
Reusable	1144 (47.4%)	
Disposable	640 (26.5%)	
Indifferent	461 (19.2%)	
I do not have experience	166 (6.9%)	
Please answer yes if you have used these devices/techniques	Yes; n (%)	<0.001
Methacrylate box with holes for arms	1832 (76%)	
Facemask sealing	2187 (90.7%)	
Negative pressure system in intubation area	1174 (48.7%)	
Avoid manual ventilation	2234 (92.7%)	
Rapid sequence induction	2373 (98.4%)	
Sellick’s maneuver	1557 (64.6%)	
Facemasck sealing with protective plastic	1664 (69%)	
In patients with suspected or positive COVID-19 diagnosis, how many clinicians experienced with airway, including you were on stage?		<0.001
One	926 (38.4%)	
Two	1299 (53.9%)	
Three or more	186 (7.7%)	
Even if you knew perfectly the sequence and approach of the airway and the preparation of the COVID-19 patient to minimize risks, do you think at any time that you had forgotten any safety steps due to the stress of the situation?		<0.001
Never	278 (11.5%)	
A few times	1677 (69.6%)	
Almost always	415 (17.2%)	
Always	41 (1.7%)	
Being in close proximity of the airway of COVID-19 positive patient, which caused more stress for you?		<0.001
Failure of intubation and patient deterioration	742 (30.8%)	
Finding an unexpected difficult airway	753 (31.2%)	
Fear of contagion	916 (38%)	

n (Number). % (Percentage). * *p* value (Pearson’s test).

## Supplementary Materials

The following supporting information can be downloaded at: https://www.mdpi.com/article/10.3390/jcm11164731/s1.
