# Supplementary material for: Airway Management of Patients with Suspected or Confirmed COVID-19: Survey Results from Physicians from 19 Countries in Latin America
Source: J Clin Med. 2022 Aug 12;11(16):4731. doi: 10.3390/jcm11164731 (PMC9410431; doi:10.3390/jcm11164731)
Supplement: Supplementary file 1 [file jcm-11-04731-s001.zip › COVID-19-SURVEY (Ingles) 37 items.pdf]

DESCRIPTIVE STUDY OF AIRWAY MANAGEMENT IN COVID19 POSITIVE PATIENTS IN LATIN AMERICA

Study code: COV2-VIAEREA

SURVEY

1. ACCEPTANCE of participation in the survey after knowing the rules of confidentiality and its voluntary participation.

DEMOGRAPHICS DATA

2. State of the Latinoamerican Federation where you work:

3. Age (number of years)

4. Which is the most accurate description of the hospital where you normally work?
    a. Public hospital
    b. Private hospital
    c. Public-private hospital
    d. Emergency extra-hospitality.

5. Seniority (years)

6. What is your medical specialty in where you have had experience with airway management?
    a. Anaesthesiology
    b. Critical care medicine
    c. Emergencies
    d. Internal medicine.
    e. Others

GENERAL CLINICAL EXPERIENCE IN COVID-19 PATIENTS

7. In your clinical practice, approximately how many patients have you intubated with a diagnosis of COVID 19?

8. In your clinical practice, approximately how many patients have you intubated with suspected COVID 19?

9. What is your medical specialty in where you have had experience with airway management in COVID-19 patients?

a. Anaesthesiology
b. Critical care medicine
c. Emergencies
d. Internal medicine.
e. Others

10. In your clinical experience, where have you intubated more COVID-19 patients?
    a. Emergencies / out-of-hospital emergencies
    b. Hospital emergencies
    c. Critical care (ICU)
    d. Urgent surgery
    e. Scheduled surgery
    f. Hospitalization room

11. Did you perform PREOXYGENATION prior to intubation / tracheostomy?
    a. Yes, for at least 5 minutes.
    b. Yes, for 3-5 minutes.
    c. Yes, for less than 1 minute.
    d. No, the patient's conditions did not allow delay
    e. No, context and the stress of the situation did not permit us to preoxygenate

12. What do you think about the following systems for the reduction of diffusion of aerosols? Rate from 1 to 6 (1: Strongly disagree, 2= Disagree, 3= Somewhat disagree, 4= Somewhat Agree, 5= Agree and 6=Strongly agree)
    a. Methacrylate box with holes for arms.
    b. Facemask sealing.
    c. Negative pressure system in intubation area.
    d. Avoid manual ventilation.
    e. Rapid sequence induction.
    f. Sellick's maneuver

13. Answer whether or not you have used these devices/techniques.
    a. Methacrylate box with arm holes: YES/NO
    b. Ordinary face mask: YES/NO
    c. Face mask with clear plastic cover: YES/NO
    d. Negative pressure system in the intubation area: YES/NO
    e. Manual ventilation avoided: YES/NO
    f. Performed Rapid Induction and Intubation Sequence (SRII): YES/NO
    g. Did you perform the Sellick Manoeuvre (cricoid pressure): YES/NO

14. As a front line exposure professional, have you ben infected by COVID-19?
    g. Yes, I have been diagnosed positive by PCR or serology.
    h. No, I have been diagnosed negative by PCR or serology.
    i. I have never been tested because I have been asymptomatic.

j. I have never been tested, although I have had symptoms.

VIDEOLARYNGOSCOPES IN COVID- 19 PATIENTS

15. Based on your COVID-19 patients' experience, what is the optimal device for intubation? Rate from 1 to 6 (1: Strongly disagree, 2= Disagree, 3= Somewhat disagree, 4= Somewhat Agree, 5= Agree and 6=Strongly agree)
    a. Video laryngoscope
    b. Direct laryngoscopy
    c. Mc Coy laryngoscopy
    d. Fiberscope

16. At your usual place of work do you have the following videolaryngoscopes available?
    - C-MAC : YES/NO
    - King Vision: YES/NO
    - McGrath: YES/NO
    - Glidescope: YES/NO
    - Airtraq: YES/NO
    - Others. YES/NO

17. What type of video laryngoscopy would you prefer to intubate a COVID-19 patient? Rate from 1 to 6 (1: Strongly disagree, 2= Disagree, 3= Somewhat disagree, 4= Somewhat Agree, 5= Agree and 6=Strongly agree).
    a. C-MAC
    b. King Vision
    c. McGrath
    d. Glidescope
    e. Airtraq
    f. I prefer to use direct laryngoscopy as I have more experience and confidence

18. What is the most frequently used device for intubation in COVID-19 patients?
    a. Video laryngoscope
    b. Direct laryngoscopy
    c. Mc Coy laryngoscopy
    d. Fiberscope

19. What kind of video laryngoscope have you used most frequently for intubation in COVID-19 patients?
    a. C-MAC
    b. King Vision
    c. McGrath
    d. Glidescope
    e. Airtraq
    f. I don't have a video laryngoscope

g. I prefer to use direct laryngoscopy with which I have more experience and confidence.

20. What disadvantages of video laryngoscopes consider the most detrimental:
    Rate from 1 to 6 (1: Strongly disagree, 2= Disagree, 3= Somewhat disagree, 4= Somewhat Agree, 5= Agree and 6=Strongly agree).
    a. Annoying light reflections on the video laryngoscope screen.
    b. Difficulty introducing into the mouth.
    c. Need for proximity to the patient's upper airways.
    d. Difficulty inserting the tube through the vocal cords.
    e. Lack of practical experience with any of the video laryngoscopes used.

21. In COVID 19 patients, what type of video laryngoscope blade do you prefer to use?
    a. Reusable
    b. Disposable
    c. Indifferent
    d. I don't have experience

22. In COVID-19 patients, what type of video laryngoscope blade do you use most frequently?
    a. Reusable
    b. Disposable
    c. Indifferent
    d. I don't have experience

23. What type of video laryngoscope blade do you prefer to use?
    a. With channel
    b. Without channel
    c. Indifferent
    d. I don't have experience

24. In COVID-19 patients, what type of video laryngoscope blade do you prefer to use?
    a. Macintosh blade
    b. Hipercurved blade
    c. Indifferent
    d. I don't have experience

25. In COVID 19 patients, what type of video laryngoscope image display monitor do you prefer to use?
    a. Video laryngoscope attachment
    b. Separate/remote from video laryngoscope
    c. Indifferent
    d. I don't have experience

DIFFICULT AIRWAY IN THE COVID-19 PATIENTS

26. What type of device do you usually use to facilitate intubation?
    Rate from 1 to 6 (1: Strongly disagree, 2= Disagree, 3= Somewhat disagree, 4= Somewhat Agree, 5= Agree and 6=Strongly agree).
    a. Frova guide
    b. Eschmann guide
    c. Flexible stylet inside the orotracheal tube.
    d. Fiberscope
    e. VAMA
    f. None

27. In the case of a predicted or known difficult airway of a COVID-19 patient, how do you prefer to perform the intubation? Rate from 1 to 6 (1: Strongly disagree, 2= Disagree, 3= Somewhat disagree, 4= Somewhat Agree, 5= Agree and 6=Strongly agree).
    a. Fiberscope
    b. Video-laryngoscope
    c. Direct laryngoscopy
    d. McCoy laryngoscopy
    e. Intubation through laryngeal mask
    f. Laryngeal mask only
    g. Tracheostomy

28. In the case of unpredicted or unknown difficult airway of COVID-19 patient, how do you prefer to perform the intubation? Rate from 1 to 6 (1: Strongly disagree, 2= Disagree, 3= Somewhat disagree, 4= Somewhat Agree, 5= Agree and 6=Strongly agree).
    a. Fiberscope
    b. Video laryngoscope
    c. Direct laryngoscopy
    d. Mc Coy laryngoscopy
    e. Intubation through laryngeal mask
    f. Laryngeal mask only

SAFETY ASPECTS FOR PROFESSIONALS

29. In postoperative COVID-19 patients who require subsequent admission to ICU, where do you prefer to extubate patients in order to minimize risk of contagion? Rate from 1 to 6. (1: Strongly disagree, 2= Disagree, 3= Somewhat disagree, 4= Somewhat Agree, 5= Agree and 6=Strongly agree).
    a. Extubating all patients in ICU
    b. Extubating all patients in the operating room.
    c. Extubating all patients in the operating room if stable and have no need for postoperative ventilation.

30. In intubated COVID-19 patients who requiring tracheostomy, consider that you agree to the following:

a. It is better to do a conventional open tracheostomy
b. It is better to do a percutaneous tracheostomy
c. It's appropriate to apply a fiberscopy to guide the optimal location in the trachea to perform open/percutaneous tracheostomy.
d. It's necessary to apply apnea/stang-by with the ventilator as much as possible in order to avoid or minimize air leakage.

31. In relation to the techniques and equipment used to intubate a COVID-19 patient, which one of the following statements do you consider important?
    a. Reuse of contaminated material is of concern to staff and patients, even if they are cleaned and disinfected.
    b. Displacement of airway equipment/devices between areas with COVID-19 patients and areas without covid patients is of concern to staff and patients.
    c. Intubation of critical or urgent COVID-19 patients is associated with more rushes for intubation and contagion risk for staff.
    d. Performing maneuvers on the airway with personal protective equipment are much more difficult and uncomfortable.

32. In relation to the equipment used to intubate a COVID-19 patient, with 1 being slightly annoying and 10 being very annoying, how annoying do you consider PPE (personal protective equipment)?

33. In relation to the PPE used to intubate a COVID-19 patient, how much do you think the added difficulty of wearing PPE was? being 1 little added difficulty, 10 much added difficulty.

34. In patients with suspected or positive COVID-19 diagnosis, How many clinics experienced with airway, including yourself, were on stage?
    a. 1
    b. 2
    c. 3 or more

35. Even if you knew perfectly the sequence and approach of the airway and the preparation of the COVID-19 patient to minimize risks, do you think at any time that you had forgotten any safety steps due to the stress of the situation?
    a. Never
    b. A few times
    c. Almost always
    d. Always

36. Being in close proximity of the airway of COVID-19 positive patient, which caused more stress for you?
    a. Failure of intubation and patient deterioration
    b. Finding an unknown difficult airway

c. Fear of contagion

37. If you have any recommendations to improve everyone's clinical practice or any information you consider important, please describe in this space.

Write your text here.
