# Supplementary material for: Airway Management of Patients with Suspected or Confirmed COVID-19: Survey Results from Physicians from 19 Countries in Latin America
Source: J Clin Med. 2022 Aug 12;11(16):4731. doi: 10.3390/jcm11164731 (PMC9410431; doi:10.3390/jcm11164731)
Supplement: Supplementary file 1 [file jcm-11-04731-s001.zip › jcm-1787507-final supplementary.pdf]

Table S1. Preferences in airway management in COVID-19 patients (continued)

Items of the questionnaire	n (%)	*p Value
In postoperative COVID-19 patients who require subsequent admission to ICU, where do you prefer to extubate patients in order to minimize risk of contagion? 6-point Likert scale [from 1 = strongly disagree to 6 = strongly agree; Percentage scoring 6: n (%)].		
Extubating all patients in ICU	937 (38.9%)	<0.001
Extubating all patients in the operating room	678 (28.1%)	<0.001
Extubating all patients in the operating room if stable and have no need for postoperative ventilation	1597 (66.2%)	0.239
In intubated COVID-19 patients who requiring tracheostomy, consider that you agree to the following? 6-point Likert scale; Percentage scoring 6: n (%).		
It is better to do a conventional open tracheostomy	531 (22%)	<0.001
It is better to do a percutaneous tracheostomy	952 (39.5%)	<0.001
It is appropriate to apply a fiberscope to guide the optimal location in the trachea to perform open/percutaneous tracheostomy	458 (19%)	<0.001
It is necessary to apply apnea/stand-by with the ventilator as much as possible in order to avoid or minimize air leakage	975 (40.4%)	0.329
In relation to the techniques and equipment used to intubate a COVID-19 patient, which one of the following statements do you consider important?. 6-point Likert scale; Percentage scoring 6: n (%).		
Reuse of contaminated material is of concern to staff and patients, even if they are cleaned and disinfected	1166 (48.4%)	<0.001
Displacement of airway equipment/devices between areas with COVID-19 patients and areas without COVID-19 patients is of concern to staff and patients	1501 (62.3%)	0.039
Intubation of critical or urgent COVID-19 patients is associated with more rushes for intubation and contagion risk for staff	1498 (62.1%)	<0.001
Performing maneuvers on the airway with personal protective equipment (PPE) are much more difficult and uncomfortable	1307 (54.2%)	<0.001
In relation to the equipment used to intubate a COVID-19 patient, with 1 being slightly annoying and 10 being very annoying, how annoying do you consider PPE?. Scored from 1 to 10, with 1 being slightly uncomfortable and 10 very uncomfortable. We take as a reference how many score 10: n (%)	519 (21.5%)	0.004
In relation to the PPE used to intubate a COVID-19 patient, how much do you think the added difficulty of wearing PPE was? Scored from 1 to 10, with 1 being little added difficulty, 10 much added difficulty. We take as a reference how many score 10: n (%)	289 (12 %)	<0.001

n (Number). % (Percentage). 6-point Likert scale [from 1 = strongly disagree to 6 = strongly agree; Percentage scoring 6: n (%)]. *P value (Pearson´s test).

Table S2. Airway expertise and most used intubation devices.

	VDL<br>n (%)	DL<br>n (%)	McCoy<br>n (%)	FBS<br>n (%)	*P value
Experts	844 (63.4%)	422 (31.7%)	64 (4.8%)	2 (0.1%)	p<0.00001
Non-experts	545 (50.6%)	475 (44.0%)	55 (5.1%)	4 (0.3%)	

n (Number). % (Percentage). VDL: Video laryngoscope, DL: Direct laryngoscopy, FBS: Fibrobronchoscopic intubation. *P value (Chi-Square analysis).

Table S3. Airway expertise and preferred intubation devices.

	VDL<br>n (%)	DL<br>n (%)	McCoy<br>n (%)	FBS<br>n (%)	*P value
Experts	915 (68.6%)	281 (21.1%)	43 (3.3%)	94 (7.1%)	p<0.81705
Non-experts	740 (68.7%)	219 (20.3%)	42 (3.9%)	77 (7.2%)	

n (Number). % (Percentage). VDL: Video laryngoscope, DL: Direct laryngoscopy, FBS: Fibrobronchoscopic intubation. *P value (Chi-Square analysis).

Table S4. Airway expertise and infection rates.

	Infected<br>n (%)	Not Infected<br>n (%)	*P value
Experts	165 (14.9%)	947 (85.1%)	p = 0.343142
Non-experts	211 (16.3%)	1088 (83.7%)	

n (Number). % (Percentage). *P value (Chi-Square analysis).

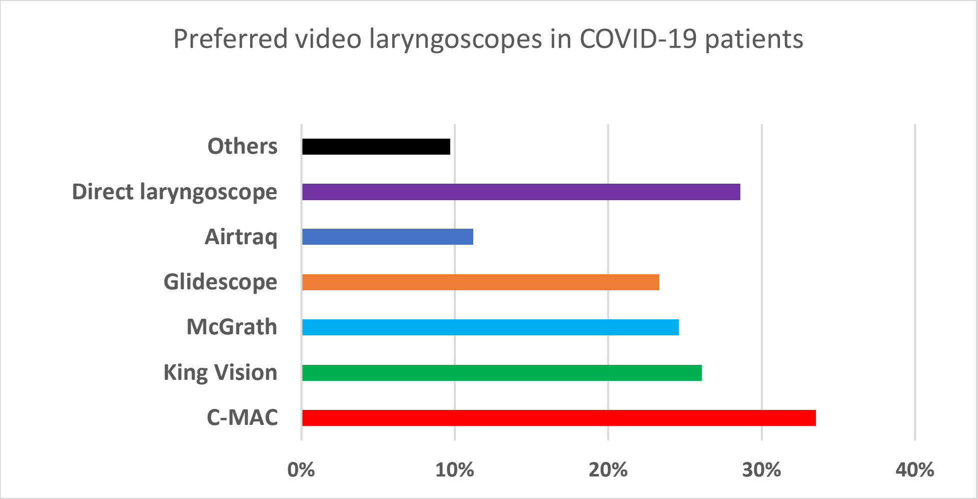

Figure S1. Preferred video laryngoscopes to intubate COVID-19 patients. The 6-point Likert scale. Percentage scoring 6 (%).

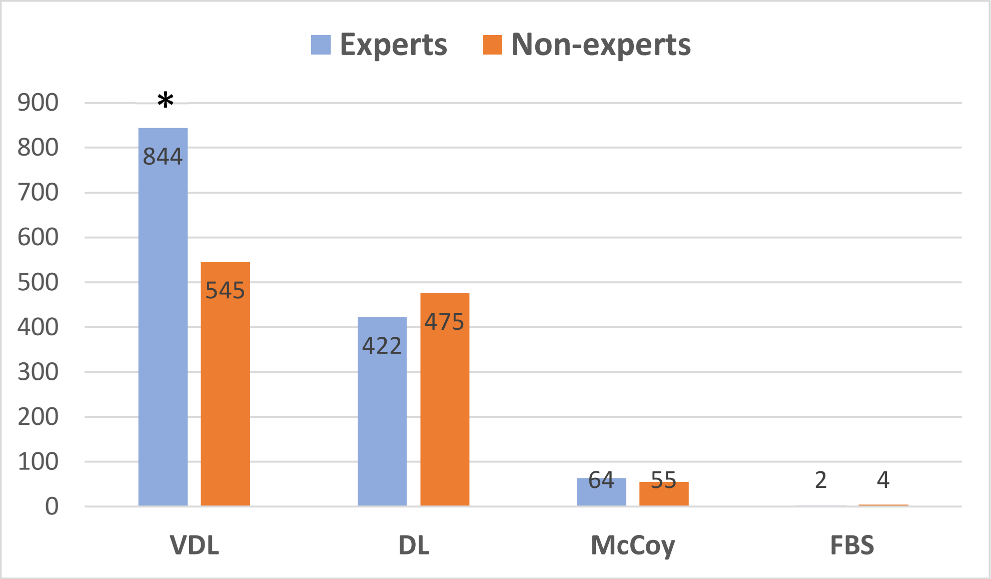

Figure S2. Airway expertise and most used intubation devices. *VDL: Video laryngoscope, DL: Direct laryngoscopy, FBS: Fibrobroncho-scopic intubation.* X^2 (1, N = 2411) =22.42, * p **< 0.001**

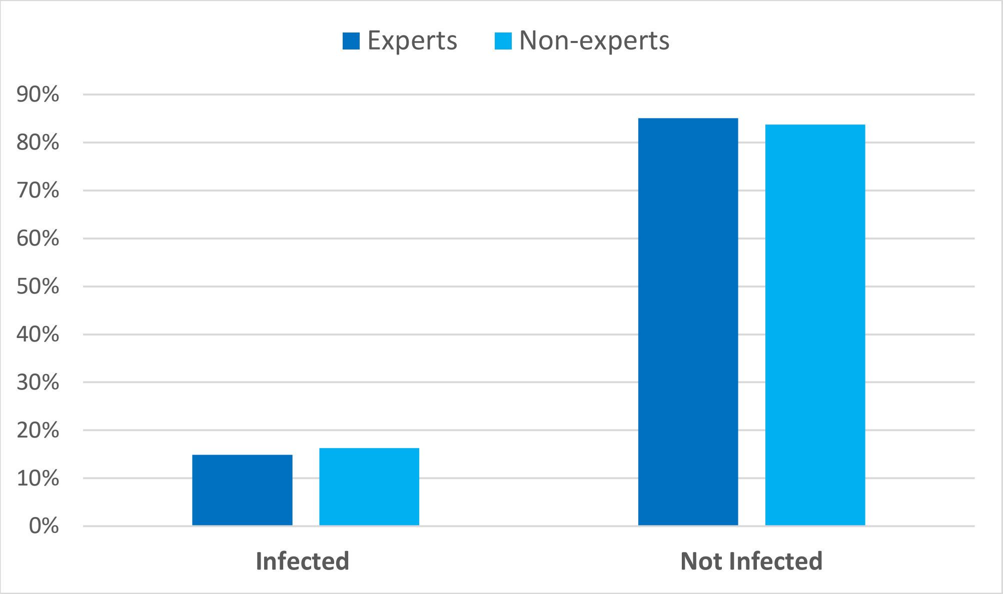

Figure S3. Airway expertise and infection rates. X^2 (1, N = 2411) = 2.53, p = 0.112.
